# Research for Prevention of Oral/Dental Diseases: How Far Have We Come?

**DOI:** 10.1177/0022034519889054

**Published:** 2019-12-20

**Authors:** F. Schwendicke, W.V. Giannobile

**Affiliations:** 1 Department of Operative and Preventive Dentistry, Charité–Universitätsmedizin Berlin, Berlin, Germany; 2 Department of Periodontics and Oral Medicine, School of Dentistry, Department of Biomedical Engineering and Biointerfaces Institute, College of Engineering, University of Michigan, Ann Arbor, MI, USA

**Keywords:** access to care, dental public health, epidemiology, health services research, preventive dentistry, socioeconomic factors

The recent *Lancet* series on oral health has wonderfully highlighted the limitations of the currently widely established dental service provision model and has recommended approaches on how to tackle these health burdens (Peres et al. 2019; Watt et al. 2019). In this month’s issue of the *Journal of Dental Research*, we highlight some of these challenges affecting dental health care delivery. The editors are grateful to Richard Watt and his colleagues as the architects of this valued series to advance awareness and continued research addressing global oral health disparities. Their team provides important implications of the work in their corresponding piece on oral and dental research and policy implications (Moynihan et al. 2020; Watt et al. 2020). With dental diseases being among the most prevalent worldwide and with overall treatment burden growing, especially in low- and middle-income countries (LMICs; Kassebaum et al. 2017), there is great need to proceed in a timely fashion to deliver actionable evidence and policies to improve oral health care globally. Universal health coverage, specifically dental, tackling the epidemic of noncommunicable diseases and the dental ones among them is critically necessary.

## The Profession’s Understanding of Itself and Its Health Care Delivery

Dentistry continues to largely focus on operative, restorative, and/or surgical interventions, often in conjunction with the usage of technological products or devices. Progress in dentistry is often defined along technological advances facilitating these reconstructive procedures, while the biological underpinnings are not as well aligned. Many dental practitioners focus on treating the symptoms of dental noncommunicable diseases (e.g., caries, periodontitis) versus emphasizing prevention and less invasive therapies more comprehensively, systematically, and interprofessionally. This clinical paradigm stems from existing dental educational models with a greater emphasis in some schools on the technical principles without similar attention paid to prevention of disease and how it can be applied in the dental practice setting. A recent review highlights the historical understanding and evolution of the dental profession based on the surgical model, as well as, importantly, the organizational structure of dental health care in most countries worldwide (Innes et al. 2019).

## Health Care Organization

The current model of providing dental services to the majority of patients has been coined “unaffordable and inappropriate”; its focus on treatment of disease instead of health maintenance has failed (Peres et al. 2019; Watt et al. 2019). While, admittedly, this model can deliver predictable therapies to provide dental function and aesthetics by advances in reconstructive therapies, it is unaffordable for many individuals due to the high financial barriers associated with complex dental reconstructions. Especially in LMICs, this model cannot be implemented or sustained given cost restraints, and it may lead to significant barriers to access for a vast share of the population. A different approach to oral and dental care is needed, and provider incentives should reflect the quality of care and the resulting patient’s oral health and overall well-being instead of the number of interventions provided. Unfortunately, there is insufficient evidence supporting different remuneration and incentive structures for quality health care improvement in dentistry (Brocklehurst et al. 2013).

## Resulting Access and Inequality

The access to dental care is limited not only in LMICs but also in high-income countries, especially for high-need populations where major social gradients exist. Moreover, prevention is mainly delivered in the dental chair, which perpetuates the problems of the interventionist care model: The dentist is responsible for “delivering” prevention; patients are “consumers” rather than participants in this preventive approach; the barriers to dental care restrict utilization of dental prevention to those who attend the practice and overcome the described financial barriers. With advances in personalized/precision medicine, the key tenets of the concept of its implementation of preventive, predictive, personalized, and participatory are critical and should be better emphasized in oral health care (Giannobile et al. 2013).

The current model of dental care and the conventional focus of “preventive dentistry” unfortunately target only a fraction of the structural and procedural causes leading to these oral health inequalities (Figure). Current dental prevention is delivered largely to those who may not necessarily need it the most. Widespread dental prevention efforts need to examine the myriad causes of the lack of penetration of prevention to broad patient populations where access to dental care is limited. One area of focus on the risk factors that dentistry shares with other noncommunicable diseases, such as free sugar, tobacco, and alcohol, should be developed, tested, and supported by dental research and dentists as well as dental organizations worldwide (Watt and Sheiham 2012; Moynihan et al. 2020). Dentistry can advocate oral health–friendly regulation and legislation, with the underlying sociobehavioral component being accounted for in current oral health care delivery systems.

**Figure.**
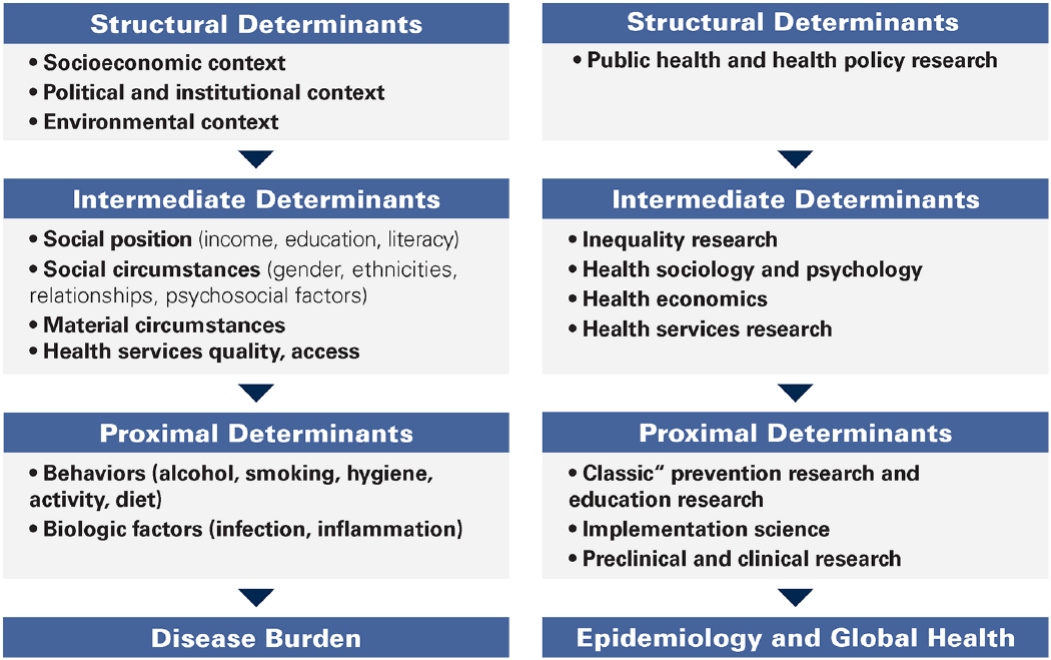
The pathway to (unfair) disease distribution (left) and research levels (right) suited to address the underlying structural or procedural causes (adapted from Watt and Sheiham 2012).

## Recommendations for the Role of Dental Research and Education

From the outlined limitations of existing care organization and delivery, a number of oral health research priorities can be recommended based on *The Lancet* Oral Health Series and current evidence.

First, there is great need for functional global oral health surveillance and health system performance measures. Contemporary health and performance indicators are imprecise or narrow; they usually reflect only specific aspects of oral health, mainly from the perspective of specific clinical disciplines, and are insufficient to comprehensively assess the impact of dental care and specific services (Watt et al. 2020). Benchmarking health care systems, comparing and thereby informing policy makers, is currently not reliably or validly possible. Refining the methodologies implemented within the Global Burden of Disease studies toward better understanding global oral health systems is needed, and expanding them from global to regional and individual health measures and performance measures is advisable (Barber et al. 2017).

Second, such an approach of better identifying the key factors would allow the steering of oral health research toward a stronger interaction with health research policy. There is great need to better prioritize research-policy interactions, with most policy being often disconnected from research findings, mainly as these are not necessarily actionable.

Third, dental research should develop and test integrative care models that are responsive to oral health needs. Having the outlined measures of quality and impact would allow us to evaluate specific interventions based on effectiveness and costs, with an improved evidence base justifying the commission or introduction of specific payment/reimbursement systems.

Finally, refining workforce and health care planning research will identify where the greatest oral health needs can be met. Research into how the dental workforce can best address the complexity in needs while accounting for the limited financial resources available is an important priority moving forward. Research and not only policy should be responsible to develop the outlined care models and associated requirements of future dental professionals. We require tools for predicting service needs and workforce supply, as well as interventions to steer workforce allocation to limit the described inequalities in health and barriers to care. The IADR’s GOHIRA initiative (Global Oral Health Inequalities: Task Group) is one that should continue to be cultivated to continue to address global oral health inequalities (Sheiham et al. 2011). The future of our dental profession can benefit by these important strategic directions!
